# Interventions for self-harm and suicidality in paediatric emergency departments: a meta-review

**DOI:** 10.1007/s00787-025-02706-9

**Published:** 2025-04-05

**Authors:** Marcela Radunz, Catherine Johnson, Bridianne O’Dea, Tracey D. Wade

**Affiliations:** Flinders University Institute for Mental Health and Wellbeing, Adelaide, Australia

**Keywords:** Emergency department, Self-harm, Suicide, Youth, Intervention

## Abstract

Paediatric emergency department (ED) presentations for self-harm and suicidality have significantly increased worldwide in the past decade, making paediatric EDs a key point of contact for young people experiencing suicidal ideation. Since 2022, four systematic reviews have been conducted on interventions for self-harm/suicidality in paediatric EDs, but findings were limited by small sample sizes and high heterogeneity. This meta-review provides recommendations to guide clinical practice and future research to enhance the quality of interventions in paediatric EDs for addressing self-harm and suicide related behaviours. Of 286 studies identified, five reviews synthesising 14 individual studies on ED-based interventions published up to May 2022 were included. Key themes and conclusions were synthesised. Three main themes emerged including: *lack of informative trials*, *low levels of intervention effectiveness* and *common intervention elements*. Informativeness of prior trials was limited by small sample sizes, lack of globally relevant research and limited stakeholder perspectives. Common intervention elements included: follow-up contact post-ED discharge, family involvement and psychoeducation with safety planning. Limited progress has been made in this field, likely due to challenges in conducting rigorous trials in paediatric EDs. Research has failed to incorporate voices of young people and their families, crucial for meeting their needs. Future research must prioritise co-design with youth, parents, and stakeholders as a critical next step in developing more effective paediatric ED interventions. Digital tools may offer promise for delivering interventions in the ED but should complement face-to-face professional contact.

Since the COVID-19 pandemic, paediatric emergency department (ED) presentations for suicide-related behaviours have significantly increased worldwide, particularly among girls and older adolescents aged 13–16 years [1–3]. Since 2001, suicide-related deaths in Australia have increased by 16.5% among young people aged 15–17, making suicide the leading cause of death in children and adolescents [4]. In Australia, youth population data showed similar results with 47.1% increase per annum since COVID-19 in New South Wales from 290 per 10,000 presentations in 2019 to 466 per 10,000 presentations in 2021 [5]. Furthermore, monthly hospital admissions for deliberate self-harm in Australian adolescents doubled between March 2020 and August 2020 and remained high [6]. This rapid increase substantiates the need for greater examination of the role of ED care for self-harm in youth.

Paediatric EDs are a key point of contact for young people who self-harm and/or are experiencing suicidal ideation [7]. EDs provide emergency management of suicide-related crises that is focused on preserving patients’ safety while also facilitating further specialist care through appropriate referrals. The potential of EDs for contributing to better outcomes for children and young people with suicidality is reflected in the increased focus on evidence-based ED initiated interventions in the academic literature. In 2010, Newton et al. [8] conducted a systematic review of ED interventions for paediatric suicide-related presentations. Of the 10 studies identified, only one focused on an ED initiated intervention. Since 2022, four reviews have been published on interventions for suicide and self-harm, including three reviews focused solely on interventions initiated in paediatric EDs [9–11] and one review including both adolescent and adult studies [12]. Despite an escalation of reviews, there remain low numbers of trials with high heterogeneity, across many different types of interventions. The limited number of published trials is likely influenced by the challenging and complex nature of EDs; busy physical spaces characterised by overcrowding, high noise levels, and constant flux between patients and providers [13–14]. Only half of those who present to EDs for self-harm presentations are treated by mental health staff [15]. Unsurprisingly, many young people have reported that their experiences in EDs for mental health support have exacerbated their distress, failed to meet their care needs, and left them feeling like a burden or undeserving of treatment [7]. Parents also report high levels of distress and feel ill-equipped to manage and respond to self-harm and suicidal ideation [16]. Parents play a crucial role in identifying self-harm behaviours and supporting their child to seek help but have reported a lack of support from services [17], an overall negative experience with service providers when trying to access support [18], as well as leaving the paediatric ED without a clear plan or knowledge on how to support their child.

This past body of evidence has informed the *Australian National Suicide Prevention Strategy 2020–*2023, which has concluded that EDs are not best placed to care for a person in suicidal distress [19]. Rather, the focus of suicide prevention efforts should be within a broad systems-based approach, integrating evidence-informed treatment across sectors, ranging from community mental health to EDs and inpatient treatments [19]. However, EDs remain central to acute care for a suicide-related crisis in children and adolescents in Australia and other countries, even though there is a clear mismatch between the needs of young people who present to the ED for self-harm and suicidal ideation, and the care they receive in EDs [20].

Although academic reviews of suicide-related studies have grown rapidly, the heterogenous nature of the studies included has precluded the conduct of meta-analyses in this field. As such, there are few definitive evidence-based guidelines on best practice for suicide-related ED-based interventions for children and adolescents. To address this gap, the present study conducts a meta-review with the following aims: (1) to synthesise findings from existing systematic reviews on paediatric ED-based psychological interventions for self-harm and suicide-related behaviours, (2) to provide comprehensive recommendations to guide future research to enhance the quality of interventions in paediatric EDs.

## Method

### Search strategy and selection criteria

A meta-review was determined as the most appropriate approach to achieve the stated aim of our study and given the heterogeneity of studies precludes a meta-analysis. Meta-reviews, often described as ‘a review of reviews’, synthesise existing systematic reviews and meta-analyses to provide a higher-level summary of evidence, helping to inform research, practice and policy [21]. The present study was conducted and reported in line with the evidence-based guidelines for reporting systematic reviews and meta-analyses [22–23] and was registered in the Open Science Framework Registries (https://osf.io/n28sh) on the 8th of April 2024. The databases PsycINFO, Medline, and Scopus were searched for relevant papers using the following search terms in the title only: (1) emergency or hospital or medical or “urgent care” or “crisis intervention” or “emergency department” and (2) suicide or suicide* or “attempted suicide” or suicidality or “suicidal ideation” or suicide attempted” or “self-harm” or “self-injur*” or “thoughts of death” or “suicidal thoughts” or “ending own” or “taking own” and (3) review or systematic or meta-analy*.

A search validation procedure was conducted in June 2024 to ensure that the search strategy employed captured the relevant reviews identified in our initial literature review of this field. A full search of all databases was then conducted from all years through to 15th of July 2024. Reviews that met the following criteria were included: (1) peer reviewed published systematic reviews and/or meta-analyses; (2) inclusion of paediatric populations (< 18 years); (3) included papers report on psychological interventions conducted in the emergency department (4) reviews are written in any language, with results of non-English reviews not presented in our syntheses but shown in the PRISMA flow diagram. Reviews were excluded if they met the following criteria: (1) did not include papers reporting on psychological interventions (i.e., safety planning or screening only in the absence of other elements of psychological interventions, such as psychoeducation, distress tolerance or coping skills); (2) included papers report on interventions conducted in an outpatient setting (i.e., Child and Adolescent Mental Health Services). No restriction was placed on the date of publication.

Papers retrieved from the three electronic databases were downloaded and duplicates were subsequently removed. Two independent assessors screened study titles and abstracts (managed by Covidence) to examine whether they related broadly to the question of interest. The full texts of all remaining articles were examined by two reviewers (MR and CJ) to assess eligibility for inclusion in the syntheses. Conflicts were discussed between the two authors in consultation with senior author (TW) until a consensus was reached. For all relevant articles at full-text, forwards and backwards handsearching was conducted to identify any other relevant reviews. The inter-rater reliability measured by Cohen’s Kappa at title and abstract was 0.82 and at full-text screening 0.83, indicating strong inter-rater reliability at both screening stages.

### Data extraction and quality of evidence

One author (MR) independently extracted information required for the narrative synthesis. The following information was extracted: author, publication year, country, pooled sample size, pooled participant demographic information including mean age and gender, as well as individual studies included in the review, date of review database search and the main aims and conclusions. To synthesise the main findings of included reviews, a thematic analysis approach was employed. First, key findings were extracted and grouped into preliminary themes. These themes were then reviewed and refined in discussion with co-authors (CJ and TW), resulting in the development of overarching themes and subthemes that captured common conclusions across included reviews.

The quality of included reviews was assessed by two authors (MR and CJ) using a measurement tool for systematic reviews, the 16-item Assessment of Multiple Systematic Reviews (AMSTAR-2; [24]). Items are rated as either ‘Yes,’ ‘Partial,’ or ‘No.’ Reviews are rated with an overall confidence level: ‘high confidence’ for one non-critical weakness, ‘moderate confidence’ for more than one non-critical weakness, ‘low confidence’ for one critical weakness, and ‘critically low confidence’ for more than one critical weakness [25]. Critical weaknesses are identified in the following items: Item 2 (availability of a review protocol), Item 4 (comprehensive literature search), Item 7 (list of excluded studies), Item 9 (risk of bias assessment), Item 11 (application of appropriate meta-analytical methods), Item 13 (discussion of risk of bias), and Item 15 (assessment of publication bias).

## Results

### Description of included reviews

**Fig. 1:**
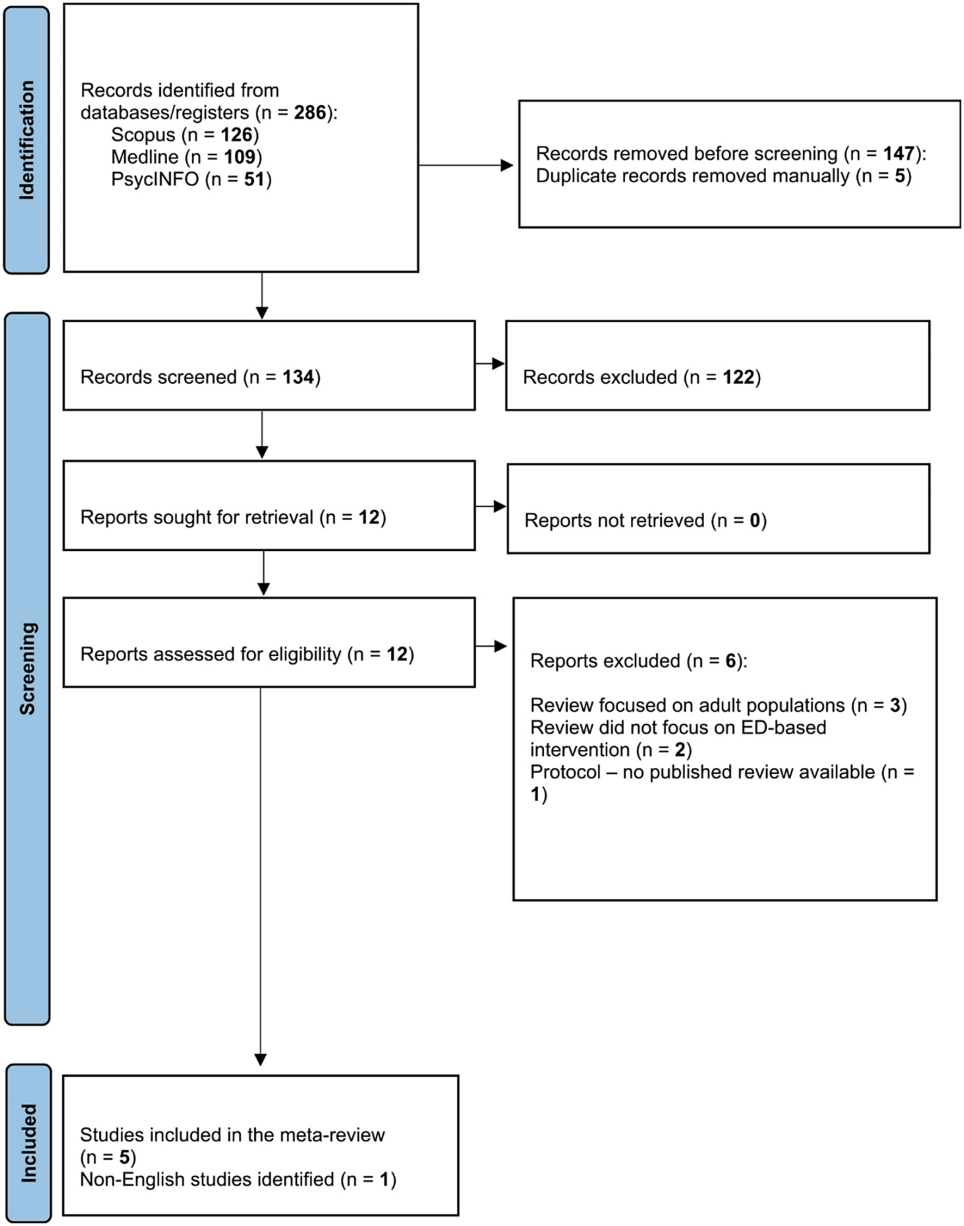
PRISMA flow diagram for scoping review

The database searches, conducted on 15th of July 2024, identified 286 studies. After removal of duplicates, 134 studies were assessed for eligibility at title and abstract screening, with a total of 12 full text articles assessed for eligibility (Fig. 1). A total of 6 reviews were included in the present meta-review. However, one of these reviews [26] met inclusion criteria, but was unable to be included in our narrative synthesis as its full text was published in French. Across the five included reviews, there were a range of interventions synthesised, including brief single session interventions delivered in the ED (i.e., ED-based interventions), post-discharge interventions (i.e., outpatient or inpatient interventions) and ED transition interventions (i.e., intervention initiated in the ED, linking patient to longer term outpatient treatment). In the current meta-review, we focus on ED-based interventions, which involve brief contact that occurs during the ED encounter.

The final sample for the meta-review synthesis consisted of five systematic reviews synthesising 14 individual studies on ED-based interventions published up to May 2022. Included reviews were conducted in USA (*n* = 1), Canada (*n* = 3) and United Kingdom (*n* = 1). Table 1 provides a detailed description of included reviews.

**Table 1:** Description of included reviews

Author (Country)	*N* Included paediatric ED Studies/Methodology of studies	Paediatric ED delivered studies identified	Date of database searches	M^age^ (range) of participants; Gender	*N* of participants included in review
Balasa et al. (2023) (Canada)	*N* = 5 (all included studies were paediatric ED-based interventions); RCTs only	Asarnow et al. (2011); Grupp-Phelan et al. (2019); King et al., (2012); King et al., (2015); Wharff et al. (2019)	All years – May 2022	Age range = 10–18 years; Gender = NR	*N* = 780
Chartier et al. (2023)^*^ (Canada)	*N* = 6 (adolescent ED-based interventions); RCTs (*n* = 4), controlled trials (*n* = 2)	Rotheram-Borus et al. (1996); Asarnow et al., (2011); Hughes et al., (2013); Wharff et al., (2012; 2019); Ougrin et al. (2013)	All years – March 2021	M^age^ = 15 years; 77.66% = female	*N* = 599
Newton et al. (2010) (Canada)	*N* = 10 (ED-based intervention = 1; post-discharge delivery = 6; ED transition interventions = 3); RCTs (*n* = 7) and quasi-experimental (*n* = 3) studies	Rotheram-Borus et al. (2000)	1985- October 2009	Age range = 10–18 Years; 72% = Female	NR
Pitts et al. (2024)^#^ (USA)	*N* = 8 (ED-based interventions)	Asarnow et al. (2011); Grupp-Phelan et al., (2019); Grupp-Phelan et al., (2012); King et al., (2015); Rotheram-Borus et al. (2000); Wharff et al., (2012); Wharff et al. (2019); Wintersteen et al. (2013)	All years – November 2020	Age range = 10–24 years; 82.7% = female	*N* = 57,291
Virk et al. (2022) (United Kingdom)	*N* = 6 (all included studies were paediatric ED-based interventions); RCTs only	Asarnow et al. (2011); Diamond et al. (2010); Grupp-Phelan et al. (2019); Hughes and Asarnow (2013); King et al. (2015); Wharff et al. (2017)	2010 – December 2020	Age range = 10–19; Gender = NR	*N* = 606

Note. # Included both paediatric and adult populations in review; *included studies on adolescent and military veteran populations

### Synthesis of key findings

The main themes and conclusions from the included reviews are summarized in Table 2. In total, three themes were identified (lack of informative trials, low levels of intervention effectiveness and common intervention elements) across ten conclusions.

**Table 2:** Summary of main themes identified across the included reviews (*n* = 5)

Main themes identified	Balasa et al. (2023)	Chartier et al. (2023)	Newton et al. (2010)	Pitts et al. (2024)	Virk et al. (2022)	Total
*Research related recommendations*						
*Need for high-quality research*	✓	NR	✓	✓	✓	4
*High heterogeneity among studies*	✓	NR	✓	NR	✓	3
*Need for clear best-practice guidelines*	✓	✓	NR	NR	NR	2
*Co-design with children and young people*	✓	NR	NR	NR	✓	2
*Conclusions regarding intervention effectiveness*						
*Increase linkage to follow-up outpatient care*	✓	✓	NR	✓	NR	3
*Reductions in depression*	✓	NR	NR	✓	✓	3
*Null effects on suicide attempts or suicide related outcomes*	✓	NR	✓	✓	NR	3
*Common intervention elements*						
*Use of follow-up contact post-ED discharge*	✓	✓	NR	NR	✓	3
*Family-Based Interventions*	✓	NR	NR	NR	✓	2
*Psychoeducation and safety planning*	NR	✓	NR	✓	NR	2

Note. NR = Not reported

### Lack of informative trials

This theme was generally the most discussed by all reviews. Four out of five reviews [8–12] argued for the need for more rigorous trial designs with larger sample sizes. Balasa et al. (2023) stressed the need for research that is globally relevant, such as large trials across different health systems and cultures. One review [8] highlighted the need for more consistency in intervention approaches and targets to enhance the ability of meta-analyses to provide clinical recommendations, as a range of therapeutic modalities and outcome measures had been used. High heterogeneity was observed among studies across three separate reviews [8–10], precluding the ability to conduct meta-analyses or quantitative syntheses of the literature. Balasa et al. [10] and Chartier et al. [12] emphasised the need for greater inclusion of expert and non-expert stakeholder perspectives in trials that aimed to be informative for developing best-practice guidelines. A greater need for co-designed interventions with children and young people was highlighted by two reviews [9–10].

### The effectiveness of ED interventions

Three reviews highlighted the lack of effectiveness of interventions for decreasing suicide attempts or suicide related outcomes [8, 10, 11], although three reviews [8–10] found that some interventions were effective for reducing depressive symptoms. Balasa and colleagues [10] concluded that depressive symptoms may be more sensitive to change, whereas suicide-related behaviours may be too distal and more difficult to modify with ED interventions. Similarly, Pitts et al. [11] concluded that ED interventions alone were not effective for decreasing future suicide attempts and ideation in youth. Three reviews [10–12] confirmed the effectiveness of ED interventions for increasing linkage to follow-up outpatient care.

### Common intervention elements

Among the effective approaches, three common elements emerged: (i) the inclusion of follow-up contact post-ED discharge, (ii) family-based interventions and (iii) psychoeducation with safety planning. Balasa and colleagues [10] concluded that telephone follow-up was an essential element of effective interventions, with all effective interventions identified in their review incorporating follow-up contact. Virk et al. [9] found that optimal engagement in ED interventions occurred when follow-up contact was initiated within 7 days of discharge whereas Chartier and colleagues [12] found positive effects with follow-ups of up to one month post-ED discharge.

The potential effectiveness of family-based interventions was emphasized by two reviews. Virk et al. [9] concluded that family-based interventions may be associated with reduction in suicidal ideation in comparison to motivational interviewing. It was argued that the effectiveness of family-based interventions was driven by strengthening family support and connection. It was recognized, however, that family-based interventions may not always be appropriate, thus paediatric EDs must employ a range of interventions. Balasa et al. [10] concluded that family-based interventions, involving both youth and their parents/caregivers should be considered for the ED context. Lastly, the importance of a collaborative approach, with particular attention to psychoeducation and safety planning, was emphasized by Chartier and colleagues [12] and Pitts et al. [11]. They reinforced the need for a structured approach to engage high-risk patients in safety planning and ensure that they have access to evidence-based services beyond the ED.

### Quality assessment

Quality assessment using the AMSTAR-2 tool (Table 3) revealed that the included reviews were of critically low quality (*n* = 4) and low quality (*n* = 1). The most frequently unmet critical item was Item 7, which requires the provision of a list of excluded studies with reasons; three out of five reviews did not provide this information. Item 4, concerning a comprehensive literature search, received a ‘*Partial’* rating in four reviews, while the provision of a protocol and justification for deviations (Item 2) was rated as ‘*Partial’* in two reviews and ‘*No’* in one. One review did not use an adequate method for assessing the risk of bias in individual studies (Item 9), and two reviews were rated as ‘*Partial’* for their lack of discussion of risk of bias in the results (Item 13). Finally, one review that conducted a meta-analysis did not perform an analysis of publication bias (Item 15).

**Table 3:** Quality assessment ratings for systematic reviews included in this meta-review using the AMSTAR-2 assessment tool

AMSTAR-2 Item	Balasa et al. (2023)	Chartier et al. (2023)	Newton et al. (2010)	Pitts et al. (2024)	Virk et al. (2022)
Item 1 *Research questions and inclusion criteria*	Y	P	Y	P	Y
Item 2* *Protocol and deviations*	P	P	N	Y	Y
Item 3 *Selection reasoning*	N	N	N	N	N
Item 4* *Comprehensive literature search*	P	P	Y	P	P
Item 5 *Duplicate study selection*	Y	Y	Y	Y	P
Item 6 *Duplicate Data extraction*	N	Y	N	Y	N
Item 7* *List of excluded studies with reasons*	Y	N	Y	N	N
Item 8 *Description of included studies*	Y	Y	Y	Y	Y
Item 9* *Risk of Bias assessment*	Y	Y	Y	P	Y
Item 10 *Report on sources of funding*	Y	Y	Y	Y	Y
Item 11* *Meta-Analysis methods*	N/A	N/A	N/A	Y	N/A
Item 12 *Meta-Analysis Risk of Bias*	N/A	N/A	N/A	Y	N/A
Item 13* *Risk of Bias when interpreting results*	P	P	Y	Y	Y
Item 14 *Heterogeneity discussed*	Y	Y	Y	P	Y
Item 15* *Publication Bias in quantitative synthesis*	N/A	N/A	N/A	N	N/A
Item 16 *Conflict of interest reported*	Y	Y	N	Y	Y
Review Quality High	Critically Low	Critically Low	Low confidence	Critically low	Critically low

*Note. Y* = Yes; P = Partial; N = No; N/A = Not applicable; * = denotes critical items on AMSTAR-2

## Discussion

This meta-review synthesised the key findings from five published reviews on interventions delivered in paediatric EDs designed to address self-harm and suicidality in children and adolescents. Three major themes were identified in our synthesis: (i) the lack of informative trials, (ii) low levels of intervention effectiveness and (iii) the emergence of common intervention elements. Our quality assessment revealed that most prior reviews were also of low quality (*n* = 5). Overall, our findings indicated that the field has made limited progress over the last 12 years, which may be attributed to the complexity of EDs and the operational barriers to conducting high-quality research in these settings.

Our meta-review emphasises the need for more informative trials in paediatric EDs for self-harm and suicide related presentations. The informativeness of prior trials was mostly limited by small sample sizes, examination of different healthcare systems and cultures which impedes globally relevant research, as well as the lack of inclusion of expert and non-expert stakeholder perspectives. While not explicitly discussed in the reviews, it is likely that some of the barriers to informative trials in this area have included limited time of clinicians when treating patients, lack of resources which provide a barrier to buy-in from ED staff, and the ethical complexities of gaining informed consent in emergency crisis settings [27–28]. These challenges are further compounded by poor trial designs including a lack of appropriate comparators in randomised controlled trials and a lack of consensus on the outcomes that should be the focus of ED-initiated interventions. In addition, many prior trials have failed to produce value for the several other stakeholders influential to the success of ED initiated interventions, such as ED staff [14]. Unfavourable staff attitudes towards suicide have been found to be a barrier to implementing evidence-based interventions and related research activities in EDs [29].

Future research may benefit from adopting an implementation framework approach to help identity the barriers and facilitators of uptake and engagement to ED interventions prior to trials being conducted. Future research will be strengthened by greater use of co-design that includes important stakeholders: children, adolescents, their families and ED staff [30–32]. Despite the well-documented negative experiences of young people presenting to EDs for suicidality and self-harm [7, 33–34] only two reviews recommended co-design with youth, and with their significant others [9–10]. By involving lived experience voices in the design and evaluation of intervention elements, future research can better address these barriers and create a more supportive, responsive care environment that meets the needs of youth in distress. Co-design was also among the twelve key recommendations from *The Lancet* Commission on Self-Harm [35], emphasizing the importance of involving individuals with lived experience of self-harm in leading and contributing to the design, delivery, and evaluation of care to enhance service delivery. Preliminary research with young people with lived experience and hospital staff has confirmed the feasibility and acceptability of incorporating digital tools in the ED [36–37]. Additionally, digital mental health tools may also improve workforce capacity in an overburdened system, by not requiring hospital staff to provide evidence-based psychological care to youth in distress [37]. To date there is little research available to guide best-practice of digital service delivery in paediatric EDs.

A major challenge reported across all reviews was the lack of consensus on outcome targets and measurement for ED-initiated interventions. The reviews highlighted that most interventions were focused on improving suicide-related or mood disorder outcomes post-discharge. Few studies focussed on relieving distress during the ED visit or other emotional factors that may mediate mental health recovery. Furthermore, while a primary function of EDs is to preserve the safety of children and young people during suicidal crises, other improvements in mental health may be initiated during these visits. Given that negative hospital experiences can deter youth from seeking help during future suicide crises [38], the findings of this meta-review support the notion that EDs present an underutilised opportunity to (i) improve knowledge and awareness of effective treatments for self-harm and suicidality among children, adolescents and their families, (ii) facilitate positive help-seeking attitudes and behaviours in children, adolescents and their families, (iii) initiate the ‘micro’ changes in the proximal psychological constructs that can help to improve more distal core mental health outcomes at later times such as hopefulness, agency and feelings of respect and value.

Future research needs to focus on designing and examining the effectiveness of multi-level interventions that target individual, family, and healthcare provider outcomes to ensure high quality care as these have been found to be better than single-level interventions for improving outcomes [39]. These multi-level interventions will, however, need to be embedded with supportive care from ED staff. In their review, MacDonald and colleagues [40] found that individuals experience extensive threats to their identity in the ED as they navigate the disclosure of their suicidality, experiencing disorientation when care ranges from gentle to hostile. In contrast, ED patients have reported feeling better, less suicidal and less likely to repeat-self harm after good-quality compassionate and supportive care [41]. In addition, suicide safety planning is an intervention element supported by evidence of effectiveness for reducing suicidal behaviour in adults [42]; this finding, however, is yet to be replicated in youth.

Our meta-review provides further support for the importance of timely follow-up as an essential component of ED-initiated interventions for self-harm and suicidality, however, the nature of this follow-up remains unclear. Future research should further explore the optimal follow-up methods for engaging with families of children and youth who present with suicidality. In addition, young people have preferred digital check ins rather than phone calls, as well as the implementation of digital therapeutic content such as coping strategies, distraction, self-reflection and a chat function [36]. Furthermore, as most individuals are discharged without receiving any follow-up [43], future research should examine novel methods of ensuring follow-up of suicidal youth who present to hospital settings [44–45].

Finally, across all reviews, care for children and young people in distress was found to be most successful when integrated with family involvement [16]. Future research needs to prioritise family involvement, so that paediatric ED interventions can support not only the young person but equip families to provide better care at home. Some learnings in this area can be gleaned from the literature showing the promise of brief, online interventions that assist families in improving outcomes for their child with anorexia nervosa in the home environment [46].

## Conclusion

In summary, this meta-review synthesised findings from existing systematic reviews on paediatric ED-based psychological interventions for self-harm and suicide related behaviours, identifying four key elements of intervention commonly emphasised in the literature—safety planning, psychoeducation, follow-up post-discharge, and family involvement. However, the overall quality of the included reviews was poor, and the evidence base surrounding the four key intervention elements remains limited, reflecting broader challenges in conducting rigorous RCTs in paediatric EDs. Further systematic reviews in this area are not indicated until future research prioritises improved quality of research design and co-design with youth, parents, and stakeholders. These are critical next steps in developing more effective paediatric ED interventions. By prioritising co-design, lived experience and evidence-based approaches at the core of future research efforts, the field can move beyond the limitations of the current literature and ensure that future research is informative.

## Data Availability

No datasets were generated or analysed during the current study.
