# Correction: Interventions for self-harm and suicidality in paediatric emergency departments: a meta-review

**DOI:** 10.1007/s00787-025-02773-y

**Published:** 2025-07-01

**Authors:** Marcela Radunz, Catherine Johnson, Bridianne O’Dea, Tracey D. Wade

**Affiliations:** Flinders University Institute for Mental Health and Wellbeing, Adelaide, Australia

**European Child & Adolescent Psychiatry**

10.1007/s00787-025-02706-9

The Funding information section was missing from this article and should have read Open Access funding enabled and organized by CAUL and its Member Institutions. Financial support was received from the Breakthrough Mental Health Foundation (MR and BO), National Health and Medical Research Council (TDW and CJ [grant: APP2025665]), the National Health and Medical Research Council Medical Research Future Fund (BO [MRF1197249] and the Little Heroes Foundation (BO).

